# Exploratory Immunohistochemical Mapping of Mechanosensitivity-Associated Ion Channel Proteins in Terminal Glial Cells of Human Meissner and Pacinian Corpuscles

**DOI:** 10.3390/ijms27146525

**Published:** 2026-07-22

**Authors:** Irene Amigo, Yolanda García-Mesa, Patricia Cuendias, Jorge Feito, Olivia García-Suárez, Ana M. Abreu-Velez, Iván Suazo, José A. Vega

**Affiliations:** 1 Servicio de Traumatología y Cirugía Ortopédica, Hospital Universitari Son Espases, 07120 Palma de Mallorca, Spain; irene.amigo.md@gmail.com; 2 Departamento de Morfología y Biología Celular, Grupo SINPOS Sistema Nervioso Periférico y Órganos de los Sentidos, Universidad de Oviedo, 33006 Oviedo, Spain; garciamyolanda@uniovi.es (Y.G.-M.); cuendiaspatricia@uniovi.es (P.C.); jfeito@usal.es (J.F.); garciaolivia@uniovi.es (O.G.-S.); 3 ISPA Instituto de Investigación Sanitaria del Principado de Asturias, 33011 Oviedo, Spain; 4 Servicio de Anatomía Patológica, Complejo Asistencial Universitario de Salamanca, 37007 Salamanca, Spain; 5 Instituto de Investigación Biomédica de Salamanca (IBSAL), 37007 Salamanca, Spain; 6 Departamento de Biología Celular y Patología, Universidad de Salamanca, 37007 Salamanca, Spain; 7 Georgia Dermatopathology Associates, Atlanta, GA 30329, USA; abreuvelez@yahoo.com; 8 Facultad de Ciencias de la Salud, Universidad Autónoma de Chile, Providencia, Santiago 7500912, Chile; ivan.suazo@uautonoma.cl

**Keywords:** cutaneous end-organ complexes, terminal glial cells, Meissner corpuscles, Pacinian corpuscles, mechanosensitive ion channel-associated proteins, acid-sensing ion channels, transient receptor potential channels, PIEZO channels, immunohistochemistry, human glabrous skin

## Abstract

Terminal glial cells (TGCs) of cutaneous end-organ complexes have traditionally been regarded as structural and trophic elements, although they may also participate in mechanosensory processing. We performed an exploratory immunohistochemical and immunofluorescence study of mechanosensitivity-associated ion channel proteins in human Meissner and Pacinian corpuscles using archival formalin-fixed, paraffin-embedded glabrous skin from 64 donors and several anatomical sites. Immunoreactivity was assessed separately in mechanoreceptor axons and TGCs. In Meissner corpuscles, TGC-associated immunoreactivity for ASIC2 and TRPV4 was observed in subsets of evaluable corpuscles, whereas TRPA1 immunoreactivity was observed in selected specimens. PIEZO1- and PIEZO2-associated immunoreactivity was observed in subsets of corpuscles from the single available labia minora specimen. Among evaluable Pacinian corpuscles, inner-core TGC-associated immunoreactivity was observed for ASIC2, PIEZO2, TRPA1, and TRPV4. Because Pacinian corpuscles were unevenly distributed and scarce outside hand and foot skin, these findings were interpreted exclusively descriptively. To our knowledge, TRPA1 immunoreactivity has not previously been described in TGCs of human sensory corpuscles. These findings are hypothesis-generating and do not demonstrate protein function or mechanotransduction. Because this study used archival tissue and independent target validation was not performed, the results require confirmation using orthogonal antibody- or transcript-based approaches.

## 1. Introduction

Glial cells associated with cutaneous end-organ complexes (CEOCs), also known as sensory corpuscles, represent a specialized subpopulation of peripheral glia referred to as terminal glial cells (TGCs) [1,2], skin end-organ glia [3] or terminal Schwann cells [4]. In vertebrate CEOCs, the TGCs envelop the peripheral axon terminals of low-threshold mechanoreceptors from development and throughout life [5]. Their arrangement differs according to the CEOC morphotype: stacked lamellar cells in Meissner corpuscles, symmetric hemi-onion bulb lamellae in Pacinian corpuscles, and irregularly organized cells in Ruffini corpuscles [6]. Classically, TGCs have been hypothesized to play structural, supportive, and trophic functions (see [2]). However, emerging data suggest that these cells may also contribute to mechanosensory processing and to the modulation of mechanoreceptor function [7,8].

Classically, the mechanical properties of the periaxonal cells in CEOCs, as well as the differentiations on the axonal membrane, were considered necessary and sufficient to generate the receptor potential and consequently the action potential (see for review [6,9]). Thus, according to this theory, mechanotransduction was regarded as a physical mechanical process. The discovery that mechanical forces can influence proteins present in mechanoreceptors and their associated cells, including TGCs, has led to a more complex view of cutaneous mechanosensitivity. These proteins, collectively referred to as mechanosensors, include transmembrane proteins and ion channel-associated proteins [10], cytoskeletal proteins [11], and components of the extracellular matrix [12]. Mechanosensors are organized into molecular complexes that may participate in mechanosensing and mechanotransduction pathways.

Mechanically gated ion channels belong to different families, although PIEZO1 and PIEZO2 are currently regarded as the principal bona fide mechanotransducers in vertebrate mechanoreceptors. Additional ion channels have been implicated in mechanosensitivity and mechanotransduction-related pathways, including members of the transient receptor potential (TRP) superfamily, particularly TRPA1, TRPC1, TRPC6, and TRPV4, as well as members of the acid-sensing ion channel (ASIC) and epithelial sodium channel (ENaC) families [13,14,15,16]. Certain voltage-gated [17,18,19,20] and ligand-gated [21,22] channels have also been associated with tactile processing. Depending on the cellular context, these proteins may act as primary sensors, modulators, amplifiers, or downstream effectors, and their precise roles remain a matter of debate.

The canonical epithelial sodium channel is composed of α-, β-, and γ-ENaC subunits. The α-subunit provides the principal pore-forming component, whereas the β- and γ-subunits modulate channel trafficking and biophysical properties. Therefore, detection of β- or γ-ENaC immunoreactivity in the absence of α-ENaC assessment cannot be interpreted as evidence of a functional ENaC channel complex.

Because CEOCs are specialized sites for the detection of mechanical stimuli, it is reasonable to hypothesize that their cellular components contain proteins associated with mechanosensitivity. Mechanosensitive ion channel-related proteins have been detected in mechanoreceptor axons and, in some cases, in TGCs of CEOCs (see [16]). These observations support the possibility that TGCs may participate in the modulation of mechanosensory signalling [23,24,25,26], although their precise functional contribution remains unresolved.

The present study was designed to analyze the immunohistochemical and immunofluorescence distribution of mechanosensitive ion channel-associated proteins in human CEOCs from glabrous skin at different anatomical locations. We investigated proteins belonging to families implicated in mechanosensing and mechanotransduction-related pathways, including ASIC1, ASIC2, ASIC3, β- and γ-ENaC, TRPA1, TRPC1, TRPC6, TRPV4, PIEZO1, and PIEZO2. The study specifically focused on immunoreactivity for these proteins in TGCs of Meissner and Pacinian corpuscles, with the aim of improving our understanding of their potential contribution to cutaneous mechanosensitivity. The primary objective was to map TGC-associated immunoreactivity for a panel of mechanosensitivity-related proteins. Axonal immunoreactivity was analyzed as a secondary descriptive outcome.

## 2. Results

Technical negative controls showed no signal above background, whereas the selected control tissues displayed staining patterns consistent with the expected distribution of the investigated targets. These controls assessed assay performance and background staining but did not independently establish target specificity.

The study included 69 tissue samples from 64 donors; some donors contributed more than one sample. The number of evaluable Meissner and Pacinian corpuscles varied according to the anatomical site and the marker examined.

Not all marker–site combinations were evaluable because Meissner or Pacinian corpuscles were absent from some sections. This was particularly frequent in the case of Pacinian corpuscles, which were identified predominantly in hand and foot skin, were scarce in the foreskin and labia minora, and were not identified in the nipple or lips. For Meissner corpuscles, the proportion of TGC-immunoreactive corpuscles is reported descriptively for each anatomical site. Pacinian corpuscle findings are presented separately as descriptive immunolocalization observations, as detailed below.

Throughout the Results Section, “immunoreactive” or “signal detected” refers exclusively to staining above the matched-control background. “Not detected” indicates that no signal above background was observed under the experimental conditions used and should not be interpreted as evidence that the target protein was absent.

**Figure 1:**
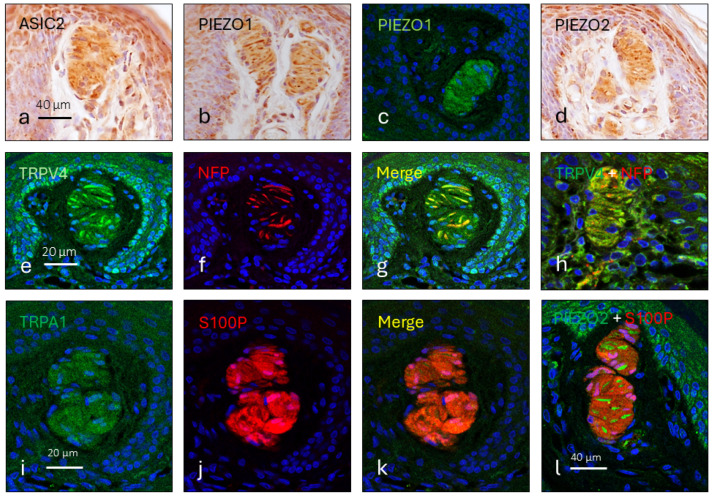
Representative sections showing the immunohistochemical and immunofluorescence detection of selected mechanosensitive ion channel-related proteins in Meissner corpuscles of human glabrous skin from different anatomical locations. (**a**) Foreskin; (**b**–**d**) labia minora; (**e**–**g**) finger; (**h**) foreskin; (**i**–**k**) toe; (**l**) finger. Counterstaining in (**a**,**b**,**d**) was performed with Mayer’s haematoxylin; blue nuclear staining in immunofluorescence images corresponds to DAPI. The scale bar in (**a**) applies to (**a**–**d**); the scale bar in (**e**) applies to (**e**–**h**); the scale bar in (**i**) applies to (**i**–**k**); and the scale bar in (**l**) applies to (**l**).

**Figure 2:**
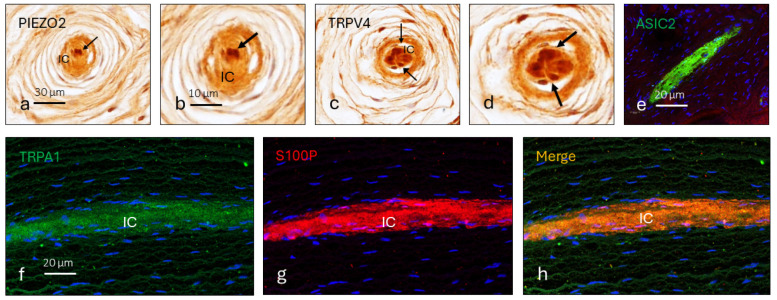
Representative sections showing the immunohistochemical and immunofluorescence detection of selected mechanosensitive ion channel-related proteins in Pacinian corpuscles of human glabrous skin from different anatomical locations. (**a**,**b**) Finger, with (**b**) representing an enlargement of (**a**); (**c**,**d**) toe, with (**d**) representing an enlargement of (**c**); (**e**) foreskin; (**f**–**h**) finger. IC: inner core. Arrows indicate axons. Counterstaining in (**a**–**d**) was performed with Mayer’s haematoxylin; blue nuclear staining in immunofluorescence images corresponds to DAPI. The scale bar in (**a**) applies to (**a**,**c**); the scale bar in (**b**) applies to (**b**,**d**); and the scale bar in (**f**) applies to (**f**–**h**).

**Figure 3:**
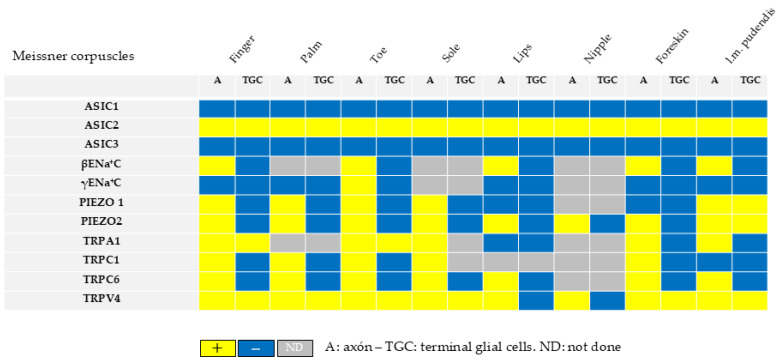
The detection of mechanosensitive ion channel-related proteins in Meissner corpuscles of human glabrous skin from different locations. Yellow indicates that immunoreactivity was detected above background, blue indicates that no signal above background was detected, and grey indicates that no evaluable corpuscles were identified in the corresponding sections.

**Figure 4:**
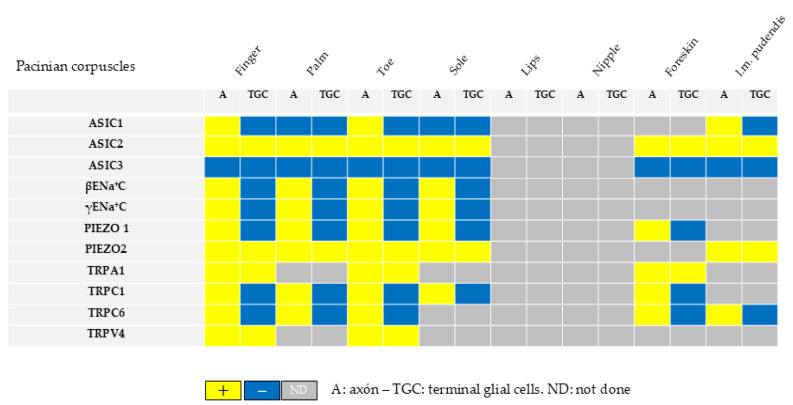
The detection of mechanosensitive ion channel-related proteins in the Pacinian corpuscles of human glabrous skin from different locations. Yellow indicates that immunoreactivity was detected above background, blue indicates that no signal above background was detected, and grey indicates that no evaluable corpuscles were identified in the corresponding sections. The findings are descriptive, and no statistical comparisons between anatomical sites were performed.

The results are illustrated by representative immunohistochemical and immunofluorescence images (Figure 1 and Figure 2) and by site-by-marker summary matrices for Meissner and Pacinian corpuscles (Figure 3 and Figure 4). Yellow indicates signal above background, blue indicates no detectable signal, and grey indicates that no evaluable corpuscles were identified.

### 2.1. Detection of Mechanosensitive Ion Channel-Associated Proteins in Mechanoreceptor Axons

The site-by-marker distribution of axonal immunoreactivity is summarized in Figure 3 and Figure 4. In Meissner corpuscles, axonal immunoreactivity was detected for ASIC2, β-ENaC, γ-ENaC, PIEZO1, PIEZO2, TRPA1, TRPC1, TRPC6, and TRPV4 in one or more evaluated anatomical sites, whereas ASIC1 and ASIC3 immunoreactivity was not detected. In Pacinian corpuscles, axonal immunoreactivity was observed for ASIC1, ASIC2, β-ENaC, γ-ENaC, PIEZO1, PIEZO2, TRPA1, TRPC1, TRPC6, and TRPV4 in one or more evaluated sites. Because α-ENaC was not assessed, β- and γ-ENaC immunoreactivity is reported only as descriptive subunit-associated staining and does not support the presence of a functional ENaC channel complex.

### 2.2. Detection of Mechanosensitive Ion Channel-Associated Proteins in Terminal Glial Cells

The immunoreactivity profile of mechanosensitive ion channel-associated proteins in sensory corpuscle TGCs was highly variable, as was the percentage of immunolabelled corpuscles across anatomical locations.

In Meissner corpuscles, ASIC2 immunoreactivity was detected in TGCs from all anatomical locations analyzed (14.2 ± 3.2% in the fingers; 8.6 ± 1.3% in the palm; 8.8 ± 2.7% in the toes; 4.1 ± 1.1% in the sole; 100% in the lips; 100% in the nipple; 17.8 ± 2.2% in the foreskin; 11.6% in the single labia minora specimen). TRPV4 immunoreactivity was observed in all locations except the lips and nipple (8.6 ± 1.2% in the fingers; 6.2 ± 1.5% in the palm; 5.7 ± 1.1% in the toes; 6.4 ± 1.9% in the sole; 14.9 ± 2.6% in the foreskin; 13.3% in the single labia minora specimen). In the single available labia minora specimen, PIEZO1 and PIEZO2 immunoreactivity was observed in 28.6% and 44.2% of evaluable Meissner corpuscles, respectively. These single-specimen observations are reported descriptively and do not support anatomical-site-specific or population-level conclusions. TRPA1 immunoreactivity was detected in fingers (18.2 ± 2.9%) and toes (13.3 ± 1.8%). The quantitative results are summarized in Table 1. ASIC1, ASIC3, β-ENaC, γ-ENaC, TRPC1, and TRPC6 immunoreactivity was not detected in TGCs of Meissner corpuscles in the anatomical locations examined (Figure 3).

A total of 31 Pacinian corpuscles were identified in the fingers, 43 in the palm, 21 in the toes, 44 in the sole, 3 in the foreskin, and 3 in the labia minora. No Pacinian corpuscles were identified in the nipple or lips (Table 2). Among evaluable Pacinian corpuscles, inner-core TGC-associated immunoreactivity was observed for ASIC2, PIEZO2, TRPA1, and TRPV4, with the anatomical distribution summarized in Figure 4.

## 3. Discussion

This exploratory descriptive study mapped immunoreactivity for selected mechanosensitivity-associated ion channel proteins in TGCs of human Meissner and Pacinian corpuscles from different anatomical sites. Although morphologically distinct, both corpuscle types contain an afferent axon surrounded by specialized TGCs and function as rapidly adapting type I and type II low-threshold mechanoreceptors, respectively [6,9]. The principal findings were TGC-associated immunoreactivity for ASIC2, TRPV4, TRPA1, and PIEZO2 in subsets of evaluable corpuscles. The observed distribution was restricted and heterogeneous and should be interpreted as anatomical mapping rather than evidence of target-specific protein expression, membrane channel activity, or regional differences in protein expression.

Although the present study focused primarily on TGCs, mechanoreceptor axons showed immunoreactivity for several of the investigated proteins, broadly consistent with previous observations in sensory neurons and cutaneous mechanoreceptors. ASIC1 and ASIC2 [16,27], ENaC subunits [28], PIEZO1 and PIEZO2 [29,30,31], TRPA1 [32], TRPC1 and TRPC6 [33,34], and TRPV4 [35] have previously been detected in sensory neurons or mechanoreceptor axons. Furthermore, all the investigated proteins apart from TRPA1 have previously been detected in the axons of different CEOC morphotypes in several animal species, including humans [16]. However, β- and γ-ENaC immunoreactivity was assessed without concomitant evaluation of the pore-forming α-ENaC subunit. Accordingly, these findings indicate only immunoreactivity against individual auxiliary subunits and do not support the existence of a functional ENaC channel complex in mechanoreceptor axons or TGCs.

In TGCs, immunoreactivity for ASIC2, PIEZO1, PIEZO2, TRPA1, and TRPV4 was observed in Meissner corpuscles, whereas ASIC2, PIEZO2, TRPA1, and TRPV4-associated signals were observed in Pacinian corpuscles. These findings are broadly consistent with previous studies in human digital Meissner corpuscles [16,36,37] and provide preliminary anatomical observations from additional sites. ASIC2 and TRPV4 immunoreactivity has also been reported in TGCs of murine Pacinian corpuscles, although it was not detected in those of *Macaca fascicularis* [16] or in avian Herbst corpuscles, the functional equivalents of mammalian Pacinian corpuscles [38]. The heterogeneous pattern observed in the present study may reflect biological differences among corpuscles or anatomical sites, but it may also be influenced by differences in tissue preservation, epitope accessibility, and assay sensitivity. The available data do not allow these possibilities to be distinguished.

A noteworthy observation was the TRPA1-associated immunoreactivity detected in TGCs, which, to our knowledge, has not previously been reported in human sensory corpuscles and therefore deserves specific consideration. TRPA1 is generally associated with polymodal nociceptive, chemical, inflammatory, and thermal signalling rather than with classical low-threshold touch transduction. Its immunoreactivity in TGCs should therefore not be interpreted as evidence that TRPA1 acts as the primary mechanically gated channel in Meissner or Pacinian corpuscles. A possible, although currently unproven, explanation is that TRPA1 participates in non-canonical glial signalling, including calcium-dependent responses, the release of paracrine mediators, or regulation of the ionic and metabolic microenvironment surrounding the afferent terminal. It might therefore modulate corpuscle excitability or adaptation rather than directly generate the receptor potential. These possibilities remain speculative and require independent confirmation of target identity and functional investigation.

Most functional evidence concerning TGCs has been obtained from Pacinian corpuscles because of their relatively large size and greater accessibility for experimental manipulation. TGCs contribute to the maintenance of the ionic and metabolic microenvironment within intercellular and interlamellar spaces and have been implicated in rapid adaptation [39,40,41,42]. Experimental studies in other models further suggest that TGCs are mechanosensitive and excitable and may influence afferent mechanosensory signalling [43,44,45,46,47]. TGC–axon interactions and mechanical anchoring may facilitate the activation of axonal PIEZO2 [23,24,25], whereas high-frequency vibration detection and stimulus-velocity sensitivity appear to depend predominantly on PIEZO2 located in the axon terminal [46]. PIEZO channels have also been implicated in myelination [48], although this function is unlikely to apply directly to the non-myelinating TGCs of sensory corpuscles. PIEZO1 and PIEZO2 have been detected in satellite glial cells of sensory ganglia [49,50], and PIEZO2 has also been reported in Schwann cells [47]. More broadly, specialized Schwann cell populations contribute to peripheral mechanosensory signalling [23,51]. These previous findings provide a plausible biological context for the present observations, but our data do not demonstrate that the detected proteins have an active role in mechanotransduction or TGC physiology.

PIEZO1- and PIEZO2-associated immunoreactivity in Meissner corpuscles from the labia minora requires particularly cautious interpretation. These signals were observed in subsets of corpuscles from the single available specimen. Because all observations from this anatomical site derive from one donor, they cannot support conclusions regarding anatomical-site specificity, regional specialization, female sexual mechanosensitivity, or population-level expression. They are therefore presented solely as preliminary, hypothesis-generating observations that require replication in a larger, independent series.

Interpretation of the immunoreactivity observed for PIEZO1, PIEZO2, TRPA1, TRPV4, and ASIC2 is also limited by the absence of independent target validation. Technical negative controls and staining patterns in selected control tissues assess assay performance and background staining but do not independently establish molecular specificity. Consequently, immunoreactivity alone should not be considered definitive evidence of target-specific protein expression, membrane localization, or functional channel activity. This consideration is particularly relevant to PIEZO1 because its immunohistochemical detection in human formalin-fixed, paraffin-embedded tissues remains technically challenging. In addition, formalin fixation may mask membrane–protein epitopes, produce false negative findings, or alter the apparent subcellular distribution of the signal. For these reasons, staining intensity was not compared between markers or anatomical sites, and the absence of detectable signal should not be interpreted as evidence that the corresponding target was absent. Future studies should confirm these observations using antibodies directed against different epitopes, peptide pre-adsorption assays, transcript-based methods such as RNAscope or in situ hybridization, Western blotting in relevant human tissues, or other orthogonal approaches.

Several additional limitations should be acknowledged. The number and distribution of evaluable corpuscles differed markedly among anatomical sites. The labia minora was represented by a single donor. Pacinian corpuscles were unevenly distributed across anatomical sites and were scarce or absent outside the hand and foot. Although the total number of Pacinian corpuscles identified at each anatomical site is reported, these counts should not be interpreted as estimates of corpuscle density or anatomical prevalence because the number of available tissue samples and evaluable sections differed substantially among sites. Moreover, the reported anatomical counts are not marker-specific denominators; therefore, the Pacinian corpuscle findings are presented exclusively as descriptive immunolocalization observations, and no statistical comparisons between anatomical sites were performed. The study was not designed or powered to assess age- or sex-related effects. Anatomical-site distribution was highly unbalanced, and sex was structurally confounded with sex-specific tissues. In addition, several marker–site combinations contained very few immunoreactive corpuscles; formal age- and sex-related inference would therefore have been unreliable. Finally, β- and γ-ENaC were assessed without the pore-forming α-ENaC subunit, precluding any conclusion regarding the presence of a functional ENaC complex.

In conclusion, this exploratory study maps immunoreactivity for selected mechanosensitivity-associated ion channel proteins in TGCs of human Meissner and Pacinian corpuscles. ASIC2-, TRPV4-, TRPA1-, and PIEZO2-associated signals were observed in subsets of evaluable corpuscles. These findings provide anatomical observations that may guide future mechanistic studies but do not independently establish target-specific protein expression, channel function, or a direct role in mechanotransduction. Independent target validation, larger and more balanced human series, and functional experiments are required.

## 4. Materials and Methods

### 4.1. Human Tissue Samples

A total of 69 glabrous skin samples from 64 donors were included in this study; some donors contributed more than one specimen. Samples were obtained from the distal phalanges of the fingers (*n* = 18), palms (*n* = 6), distal phalanges of the toes (*n* = 11), soles (*n* = 4), lips (*n* = 6), nipples (*n* = 11), labia minora (*n* = 1), and foreskin (*n* = 12). These anatomical sites may contain different types of cutaneous end-organ complexes (CEOCs), particularly Meissner and Pacinian corpuscles [6]. The samples were obtained from 33 female and 31 male donors with no documented history of neurological disease. Donor age ranged from 26 to 78 years (mean age, 57.2 years). Demographic characteristics and the anatomical distribution of the samples are summarized in Table 3.

The tissues were fixed in 10% neutral buffered formalin (in 1 M PBS, pH 7.4), routinely paraffin embedded, and cut into 10 μm thick sections perpendicular to the skin surface. Exact cold ischaemia and fixation durations were not available for all archival specimens. This pre-analytical heterogeneity was considered a potential source of variation in antigen preservation.

The tissues were obtained from the Human Tissue Bank associated with the SINPOS Research Group (Peripheral Nervous System and Sense Organs Group, University of Oviedo; National Registry of Biobanks, Collections Section; Ref. C-0001627). Their use complied with applicable Spanish legislation (RD 1301/2006, Law 14/2007, RD 1716/2011, and Order ECC/1404/2013) and with the principles of the Declaration of Helsinki. The study was approved by the Research Ethics Committee of the Principality of Asturias (CEImPA project 266/18).

### 4.2. Simple Immunohistochemistry

Sections were deparaffinized and rehydrated. All sections used for the immunohistochemical or immunofluorescence detection of ion channel-associated proteins were subjected to the same standardized heat-induced epitope retrieval protocol. Sections were incubated in EnVision™ FLEX Target Retrieval Solution, High pH (50×; Tris/EDTA, pH 9.0; Code K8004; Dako/Agilent Technologies, Glostrup, Denmark) at 65 °C for 20 min using a thermostatically controlled water bath and subsequently maintained in the same solution at room temperature for an additional 20 min. No antibody-specific epitope retrieval conditions were used. Epitope retrieval was omitted when synaptophysin or S100 protein was assessed alone. In double-immunofluorescence experiments, sections underwent the retrieval protocol required for the corresponding ion channel-associated target.

For immunohistochemistry, sections were subsequently permeabilized with 0.5% Tween-20 in 1 M PBS, pH 7.6, and endogenous peroxidase activity was blocked with 10% H_2_O_2_ for 30 min. Nonspecific binding was blocked with 25% fetal bovine serum, and the sections were incubated overnight at 4 °C in a humid chamber with the primary antibodies listed as suitable for IHC in Table 4. The sections were then washed and incubated for 90 min with anti-rabbit IgG diluted 1:500 (Sigma-Aldrich, St. Louis, MO, USA) or anti-mouse IgG diluted 1:300. After further washing in TBS-T, peroxidase activity was visualized using 3,3′-diaminobenzidine as the chromogen. Finally, the sections were counterstained with Mayer’s haematoxylin for 30 s and mounted with Entellan^®^ (Merck KGaA, Darmstadt, Germany). Images were acquired using a Nikon Eclipse^®^ 80i optical microscope coupled to a Nikon^®^ DS-5M camera (Nikon Instruments, Tokyo, Japan).

The primary antibodies used in this study are listed in Table 4. In this table, “host species” refers to the species in which the antibody was raised and does not indicate the species of the target protein. Most antibodies used in the present study were raised in rabbit or mouse against epitopes corresponding to human proteins, as specified by the manufacturers and detailed in Table 4.

The anti-PIEZO1 antibody used in this study was a rabbit polyclonal antibody from Proteintech Group, Inc. (Cat. No. 15939-1-AP, Rosemont, IL, USA), raised against a recombinant human PIEZO1/FAM38A extracellular-domain fragment corresponding to amino acids 67–373. According to the manufacturer, the antibody is reactive with human samples and is suitable for immunohistochemistry and immunofluorescence/immunocytochemistry. Because the antibody recognizes an extracellular-domain epitope, membrane permeabilization is not strictly required. However, all FFPE sections in the present study were processed using the same standardized Tween-20-containing protocol. As permeabilization may influence the apparent subcellular staining pattern, PIEZO1 immunoreactivity was interpreted as cell-associated signal and not as evidence of membrane localization or functional PIEZO1 channel activity. Independent validation using additional antibodies directed against different epitopes and/or antibody-independent approaches would further strengthen these findings.

The technical performance of the immunolabelling procedures was assessed using negative controls in which the primary antibody was omitted or replaced with non-immune rabbit or mouse serum. In addition, selected positive and negative control tissues with documented presence or absence of the investigated targets were examined, including dorsal root ganglia, intestine, liver, skeletal muscle, lung, pancreas, and skin. For immunofluorescence experiments, additional controls were performed by omitting both primary and secondary antibodies to assess tissue- and fixation-related autofluorescence. These controls assessed assay performance and background staining but did not independently establish target specificity. Orthogonal validation approaches, including additional validated antibodies, peptide pre-adsorption assays, RNAscope, in situ hybridization, or Western blotting in the same human tissues, would be required for independent confirmation of target identity.

### 4.3. Simple Immunofluorescence

Sections were deparaffinized, rehydrated, and subjected to the same standardized heat-induced epitope retrieval protocol described in Section 4.2 for the immunofluorescence detection of ion channel-associated proteins. Sections were subsequently permeabilized with 0.5% Tween-20 in 1 M PBS, pH 7.6. Nonspecific binding was blocked with 10% fetal bovine serum, and the sections were incubated overnight at 4 °C in a humid chamber with the primary antibodies listed as suitable for IF in Table 4.

The sections were then washed with PBS-T and incubated for 90 min with the appropriate secondary antibody: Alexa Fluor 488-conjugated goat anti-rabbit IgG (1:100; Serotec™, Oxford, UK) or Cy3-conjugated donkey anti-mouse IgG (1:200; Jackson ImmunoResearch™, Baltimore, MD, USA). Incubation was performed at room temperature in a dark, humid chamber.

After washing with PBS, the sections were stained with DAPI (4′,6-diamidino-2-phenylindole; 10 ng/mL) for nuclear counterstaining and mounted using diluted Fluoromount-G mounting medium (SouthernBiotech, Birmingham, AL, USA). Images were acquired using a Leica DMR-XA automated fluorescence microscope and Leica Confocal Software v2.5 (Leica Microsystems, Heidelberg, Germany) at the Image Processing Service of the University of Oviedo.

Technical controls were performed as described for immunohistochemistry. Additional controls in which both the primary and secondary antibodies were omitted were used to assess tissue- and fixation-related autofluorescence.

### 4.4. Double Immunofluorescence

Double immunofluorescence was used to identify the cellular source of ion channel-associated immunoreactivity within Meissner and Pacinian corpuscles. Ion channel-associated proteins were examined together with neuronal or glial markers identifying the main cellular components of the corpuscles, including neurofilament proteins, neuron-specific enolase, synaptophysin, and S100P [6]. Double immunofluorescence was performed exclusively for ion channel-associated proteins that showed immunoreactivity by immunohistochemistry or simple immunofluorescence.

Sections were deparaffinized, rehydrated, and subjected to the same standardized heat-induced epitope retrieval protocol described in Section 4.2. The sections were subsequently permeabilized and incubated overnight at 4 °C in a humid chamber with an antibody against the investigated ion channel-associated protein together with one of the cellular markers NFP, NSE, synaptophysin, or S100P, each at the working dilution specified in Table 4. Only primary-antibody combinations raised in different host species were used, allowing for selective detection with species-specific secondary antibodies. Accordingly, mouse-derived antibodies against β-ENaC, TRPC1, and TRPC6 were not combined with the mouse-derived cellular marker antibodies in double-immunofluorescence experiments.

The sections were then washed with PBS-T for 30 min and incubated for 90 min with Alexa Fluor 488-conjugated goat anti-rabbit IgG (1:100; Serotec™, Oxford, UK), followed by incubation for 90 min with Cy3-conjugated donkey anti-mouse IgG (1:200; Jackson ImmunoResearch™, Baltimore, MD, USA). Both incubations were performed at room temperature in a dark, humid chamber.

Following secondary-antibody incubation, nuclear counterstaining with DAPI, mounting, and image acquisition were performed as described in Section 4.3. Technical controls were performed as described for immunohistochemistry. Additional controls in which all primary and secondary antibodies were omitted were used to assess tissue- and fixation-related autofluorescence.

### 4.5. Quantitative Analyses

The percentage of Meissner corpuscles displaying immunoreactivity for the assessed mechanosensitive ion channel-related proteins was determined by analyzing two sections per skin sample, separated by 200 μm and processed for the simultaneous detection of S100P and each mechanoprotein. Five fields per section were captured at ×10 magnification using Leica Confocal Software v2.5 (Leica Microsystems GmbH, Heidelberg, Germany), and the number of Meissner corpuscles was counted by two independent observers; the results were then averaged. Meissner corpuscles displaying S100P immunoreactivity were considered the total evaluable population, and those displaying immunoreactivity for both S100P and the corresponding mechanoprotein were used to determine the percentage of immunoreactive Meissner corpuscles. Corpuscles were classified as TGC-immunoreactive only when the mechanoprotein-associated signal clearly exceeded the matched negative control background and colocalized with S100-positive TGCs. No staining-intensity grading was performed. For anatomical sites represented by multiple samples, the results are presented descriptively as the mean ± standard deviation. For the labia minora, which was represented by a single specimen, the results are presented as a single percentage without a measure of dispersion. Because Pacinian corpuscles were unevenly distributed across anatomical sites, no marker-specific percentages or inferential statistical comparisons were calculated. The total number of Pacinian corpuscles identified in the examined material was recorded descriptively for each anatomical site.

## Figures and Tables

**Table 1:** Percentage of human Meissner corpuscles showing TGC-associated immunoreactivity across anatomical sites.

	Fingers	Palm	Toes	Sole	Lips	Nipple	Foreskin	Labia Minora
**ASIC2**	14.2 ± 3.2	8.6 ± 1.3	8.8 ± 2.7	4.1 ± 1.1	100	100	17.8 ± 2.2	11.6 *
**PIEZO1**								28.6 *
**PIEZO2**								44.2 *
**TRPA1**	18.2 ± 2.9		13.3 ± 1.8					
**TRPV4**	8.6 ± 1.2	6.2 ± 1.5	5.7 ± 1.1	6.4 ± 1.9			14.9 ± 2.6	13.3 *

Values are percentages and are presented as reported in the original descriptive analysis. Where available, data are expressed as the mean ± SD. Grey cells indicate that no immunoreactivity was detected in the TGCs for the corresponding protein and anatomical region. Percentages should be interpreted in relation to the number of corpuscles available in each anatomical region; therefore, in regions with few available corpuscles, high percentages may reflect a limited number of evaluated structures. * Labia minora was represented by a single specimen from one donor; therefore, values are presented without a measure of dispersion and should be interpreted only as single-specimen descriptive observations. They do not support population- or anatomical-site-level inference. No inferential statistical comparisons were performed.

**Table 2:** Number of Pacinian corpuscles identified according to anatomical site.

Anatomical Site	Pacinian Corpuscles Identified
Fingers	31
Palm	43
Toes	21
Sole	44
Lips	0
Nipple	0
Foreskin	3
Labia minora	3

The values represent the total number of Pacinian corpuscles identified in the examined material at each anatomical site and should not be interpreted as marker-specific denominators. For marker–site combinations classified as TGC-immunoreactive in Figure 4, the inner-core TGC-associated signal was observed in at least one evaluable Pacinian corpuscle in the corresponding sections. Because marker-specific denominators were not available, the proportion of immunoreactive corpuscles could not be estimated. These findings are exclusively descriptive, and no statistical comparisons between anatomical sites were performed.

**Table 3:** Demographic characteristics of donors and anatomical distribution of tissue samples.

**Donor Demographics**			
**Age Range (Years)**	**Female (*n*)**	**Male (*n*)**	**Total (*n*)**
26–40	14	12	26
41–60	11	10	21
≥61	8	9	17
Total donors	33	31	64
**Anatomical Distribution of Tissue Samples**			
**Anatomical Site**		**Samples (*n*)**	
Finger distal phalanx		18	
Palm		6	
Toes		11	
Sole		4	
Lips		6	
Nipple		11	
Labia minora		1	
Foreskin		12	
Total samples		69	

Donor demographics refer to 64 individuals, whereas anatomical site data refer to 69 tissue samples. Some donors contributed more than one sample.

**Table 4:** Primary antibodies used in study.

Antigen	Clone	Host Species	Dilution	Supplier	Catalogue Number	Supplier- Reported Human Reactivity	IHC/IF
ASIC1	EPR25411-45	Rabbit	1:200	Abcam plc ^1^	ab284410	Yes	IF
ASIC2	-	Rabbit	1:200	LifeSpan BioSciences ^2^	LS-B156	Yes	IHC/IF
ASIC3	-	Rabbit	1:100	LifeSpan BioSciences ^2^	LS-C31243	Yes	IHC
β-ENaC	E10	Mouse	1:200	Santa Cruz Biotechnology ^3^	sc-48428	Yes	IHC
γ-ENaC	-	Rabbit	1:200	Sigma-Aldrich/Merck ^4^	HPA071194	Yes	IHC/IF
PIEZO1	-	Rabbit	1:100	Proteintech Group, Inc. ^5^	15939-1-AP	Yes	IHC/IF
PIEZO2	1H10	Rabbit	1:200	Sigma-Aldrich/Merck ^4^	ZRB1468	Yes	IHC/IF
TRPA1	EPR26211-139	Rabbit	1:100	Abcam plc ^1^	ab320715	Yes	IHC/IF
TRPC1	E6	Mouse	1:100	Santa Cruz Biotechnology ^3^	sc-133076	Yes	IHC/IF
TRPC6	-	Mouse	1:100	Novus Biologicals ^6^	NBP3-26742	Yes	IHC
TRPV4	-	Rabbit	1:200	Abcam plc ^1^	ab63003	Yes	IHC/IF
NSE	BBS/NC/VI-H14	Mouse	1:1000	Dako/Agilent ^7^	M0873	Yes	IHC/IF
NFP	2F11	Mouse	1:200	Roche Diagnostics ^8^	05267714001	Yes	IHC/IF
Synaptophysin	27G12	Mouse	1:1000	Leica Biosystems ^9^	NCL-L-SYNAP-299	Yes	IHC/IF
S100 protein	4C4.9	Mouse	1:500	Roche Diagnostics ^8^	05278104001	Yes	IHC/IF

^1^ Cambridge, UK; ^2^ Seattle, WA, USA; ^3^ Santa Cruz, CA, USA; ^4^ St. Louis, MO, USA; ^5^ Rosemont, IL, USA; ^6^ Littleton, CO, USA; ^7^ Glostrup, Denmark; ^8^ Vienna, Austria; ^9^ Madrid, Spain. IHC: immunohistochemistry; IF: immunofluorescence. ASIC1: Monoclonal antibody raised in rabbit against an epitope between amino acids 195–297 of human ASIC1. ASIC2: Polyclonal antibody raised in rabbit specifically targeting amino acids 481–530 of the extracellular domain of human ASIC2. ASIC3: Polyclonal antibody raised in rabbit against a synthetic peptide located between amino acids 53–102 of human ASIC3. β-ENaC: Monoclonal antibody raised in mouse against a synthetic epitope located between the amino acids 271–460 of human β-ENaC. γ-ENaC: Polyclonal antibody produced in rabbit against human SCNN1G/epithelial sodium channel γ subunit. PIEZO1: Polyclonal antibody raised in rabbit against a recombinant human PIEZO1/FAM38A extracellular-domain fragment corresponding to amino acids 67–373. PIEZO2: Monoclonal antibody raised in rabbit against a peptide 22 amino acids long of the extracellular domain of human PIEZO2. TRPA1: Monoclonal antibody raised in rabbit against a synthetic peptide corresponding to an internal sequence of human TRPA1/TSA. TRPC1: Monoclonal antibody raised in mouse against the amino acid sequence 689–793 of the C-terminal domain of human TRPC1. TRPC6: Monoclonal antibody raised in mouse against a full-length recombinant protein corresponding to human TRPC6. TRPV4: Polyclonal antibody raised in rabbit against a synthetic peptide corresponding to amino acids 720–769 within the intracellular C-terminal region of human TRPV4.

## Data Availability

The data presented in this study are available from the corresponding author upon reasonable request.

## Abbreviations

The following abbreviations are used in this manuscript:

ASIC	Acid-sensing ion channel
CEOCs	Cutaneous end-organ complexes
DAPI	4′,6-diamidino-2-phenylindole
ENaC	Epithelial sodium channel
β-ENaC	Beta subunit of Epithelial Sodium Channel
γ-ENaC	Gamma subunit of Epithelial Sodium Channel
IF	Immunofluorescence
IgG	Immunoglobulin G
IHC	Immunohistochemistry
NFP	Neurofilament protein
NSE	Neuron-specific enolase
PBS	Phosphate-buffered saline
PBS-T	Phosphate-buffered saline with Tween-20
PIEZO1	Piezo-type mechanosensitive ion channel component 1
PIEZO2	Piezo-type mechanosensitive ion channel component 2
S100P	S100 calcium-binding protein P
SYN	Synaptophysin
TBS-T	Tris-buffered saline with Tween-20
TGCs	Terminal glial cells
TRP	Transient receptor potential
TRPA1	Transient receptor potential ankyrin 1
TRPC1	Transient receptor potential canonical 1
TRPC6	Transient receptor potential canonical 6
TRPV4	Transient receptor potential vanilloid 4
